# Sex differences in clinical cognitive impairment with Lewy bodies: a Chinese multicenter study

**DOI:** 10.1186/s13293-022-00464-w

**Published:** 2022-10-01

**Authors:** Jinghuan Gan, Zhichao Chen, Zhihong Shi, Xudong Li, Shuai Liu, Yiming Liu, Hongcan Zhu, Lu Shen, Guili Zhang, Yong You, Qihao Guo, Nan Zhang, Yang Lv, Baozhi Gang, Junliang Yuan, Yong Ji

**Affiliations:** 1 Department of Neurology, Beijing Tiantan Hospital, Capital Medical University, China National Clinical Research Center for Neurological Diseases, No. 119 Nansihuan Xilu, Fengtai, Beijing, 100070 China; 2 Department of Neurology, Beijing Friendship Hospital, Capital Medical University, Beijing, China; 3 Department of Neurology, Tianjin Huanhu Hospital, Tianjin Key Laboratory of Cerebrovascular and of Neurodegenerative Diseases, Tianjin Dementia Institute, 6 Jizhao Road, Jinnan, Tianjin, 300350 China; 4 Department of Neurology, Qilu Hospital, Shandong University, Shandong, China; 5 Department of Neurology, The First Affiliated Hospital of Zhengzhou University, Zhengzhou, Henan China; 6 Department of Neurology, Xiangya Hospital, Central South University, Hunan, China; 7 Department of Neurology, Second Affiliated Hospital of Hainan Medical University, Haikou, China; 8 Department of Gerontology, Shanghai Jiao Tong University Affiliated Sixth People’s Hospital, Shanghai, China; 9 Department of Neurology, Tianjin Medical University General Hospital, Tianjin, China; 10 Department of Geriatrics, The First Affiliated Hospital of Chongqing Medical University, Chongqing, China; 11 Department of Neurology, The First Affiliated Hospital of Harbin Medical University, Harbin, China; 12 Department of Neurology, Peking University Sixth Hospital, Beijing, China

**Keywords:** Gender, Sex ratio, Lewy body disease, Mild cognitive impairment, Parkinson’s disease

## Abstract

**Background:**

Research on sex ratios of Lewy body dementia is controversial, established in small samples, and rarely focused on prodromal stage. The objective is to investigate the clinical sex ratios (men/women) and their associations with clinical features among individuals with mild cognitive impairment with Lewy bodies (MCI-LB), dementia with Lewy bodies (DLB), Parkinson’s disease with mild cognitive impairment (PD-MCI), and Parkinson’s disease with dementia (PDD) in China.

**Methods:**

We conducted a multicenter cohort study, including 1038 individuals with probable MCI-LB, DLB, PD-MCI, or PDD diagnosis from 22 memory clinics in China from January 2018 to March 2022, and recorded their demographic and clinical data by reviewing medical records. Descriptive and regression analyses were used to calculate the sex ratio (men/women), and its associations with demographic and clinical data.

**Results:**

In this study, men comprised 35.14% (men/women sex ratio = 0.54) for MCI-LB, 46.72% (men/women sex ratio = 0.88) for DLB, 63.56% (men/women sex ratio = 1.74) for PD-MCI, and 52.40% (men/women sex ratio = 1.10) for PDD. Sex ratios roughly increased with age. Men had more parkinsonism (*p* = 0.000) and less fluctuating cognition (*p* = 0.024) in MCI-LB, and those with PD-MCI had more RBD (*p* = 0.001). Women with PD-MCI had lower MMSE scores (*β* ± standard error =  − 1.24 ± 0.58, *p* = 0.04), more irritability (0.95 ± 0.46, *p* = 0.04) and fluctuating cognition (− 3.41 ± 1.31, *p* = 0.01), and less parkinsonism (− 2.10 ± 0.97, *p* = 0.03) than men after adjusting for demographic and cardiometabolic conditions.

**Conclusion:**

There were more women in DLB and MCI-LB, and more men in PD-MCI and PDD. The sex distribution, demographic, and clinical characteristics differed, which strengthened the independence and heterogeneity of the four diseases, and indicated sex-sensitive strategies for management of dementia necessary.

**Supplementary Information:**

The online version contains supplementary material available at 10.1186/s13293-022-00464-w.

## Highlights

- There are significant sex differences in Chinese population with cognitive impairment in Lewy body disease.
- Women were more common in dementia with Lewy bodies and mild cognitive impairment with Lewy bodies cases, had more frequent and severe neuropsychiatric symptoms, and poorer cognition than men.
- Men predominant in Parkinson’s disease with mild cognitive impairment and Parkinson’s disease with dementia cases, and performed more frequent RBD and parkinsonism than women.
- Dementia with Lewy bodies *vs.* Parkinson’s disease with dementia, and mild cognitive impairment with Lewy bodies *vs.* Parkinson’s disease with mild cognitive impairment are distinct disease forms and should not be confused.

**Supplementary Information:**

The online version contains supplementary material available at 10.1186/s13293-022-00464-w.

## Background

Lewy body dementia (LBD) comprises a Lewy body disease spectrum of dementia with Lewy bodies (DLB) and Parkinson’s disease with dementia (PDD), and is the second most common neurodegenerative dementia. The two dementias are demarcated clinically from one another by the “one-year rule”, based on the temporal onset of parkinsonism relative to cognitive impairment (i.e., in PDD the motor symptoms precede the onset of dementia by at least one year) [1]. There is considerable clinical heterogeneity; however, some overlaps with core clinical features occur in the prodromal stage [2].

The scientific literature on sex distribution in dementia traditionally reports a more pronounced prevalence and incidence of men in LBD [3, 4], with about 3:2 men/women ratios in DLB, and PDD [5, 6]. Neuropathological studies indicated that women were more predominant in the mixed pathology of “Alzheimer’s disease (AD) + DLB” than in pure LBD, with less likely neocortical (“diffuse”) or intermediate (“limbic”) Lewy body pathologies [7]. Our multicenter study of individuals with LBD in Chinese memory clinics showed a slightly male predominance (50.9% in DLB cases, 57.9% in PDD cases), but without significant sex differences [8]. However, a systemic review [9] showed that five of eight studies with the gender information reported disproportionately more women with DLB, and other three reported disproportionately more men. The slightly female predominance was also reported in a retrospective cohort, with a proportion of 51.4% in the DLB group [10]. Moreover, women with LBD were more prevalent for hallucinations [11], depression and anxiety, and sleep disorders [2] but less frequent with rapid eye movement (REM) sleep behavior disorder (RBD) [12, 13] than men. The sex ratio in LBD remains unclear, particularly in the prodromal stage, with a slight predominance of either men or women. Considering the generally low prevalence of LBD in the general population, as well as a low diagnostic accuracy with available clinical diagnostic criteria, published studies have used limited sample sizes.

To better describe the sex ratios and their relationship with clinical features in a large sample, we provide a multicenter study aiming to investigate the sex distribution among individuals diagnosed with “probable DLB, MCI-LB, PD-MCI and PDD” in China. We hypothesized that the sex differences were significant in DLB, MCI-LB, PD-MCI and PDD, and would be associated with demographic and clinical characteristics, as well as the presence of core clinical features of LBD.

## Methods

### Study design and participants

This is the second report as a part of the multicenter study on the clinical features of Lewy body disease in China carried out from January 2018 to March 2022 by the China Lewy Body Disease Collaborative Alliance (unpublished). A total of 22 memory clinics from 12 provinces (Additional file 1: eAppendix 1) participated in this multicenter study and provided 1 159 “probable cognitive impairment in Lewy body disease (including DLB, MCI-LB, PD-MCI and PDD)” medical records. After confirmation by two experienced neurologists double-blindly following the criteria, 121 patients with incomplete demographic information (*n* = 43) or classified as “uncertain diagnosis” (*n* = 78) were excluded, and 1038 patients (including 74 patients with MCI-LB, 533 with DLB, 118 with PD-MCI, and 313 with PDD) were analyzed. The overall sex ratio of cognitive impairment in Lewy body disease (men/women) was 0.88 (459/524), with an average age of 69.89 ± 8.45 years.

The inclusion criteria were (a) patients were 40–100 years of age and (b) the initial clinical diagnosis was probable MCI-LB, DLB, PD-MCI or PDD. Demographic data, cognitive status, clinical diagnosis at last visit, clinical core features [fluctuating cognition (FLC) [14], visual hallucinations (VHs) [15], parkinsonism [16], and RBD [17, 18]], and neuropsychological assessments [Mini-Mental State Examination (Chinese version) (C-MMSE) [19], Montreal Cognitive Assessment (MoCA) [20], the Activities of daily living (ADL) [21], and the Clinical Dementia Rating (CDR) [22]] were mandatory; while the Neuropsychiatric Inventory (NPI) assessment [15], magnetic resonance imaging (MRI) visual rating scales [Medial Temporal lobe Atrophy (MTA) [23] and Fazekas scales [24]], and Apolipoprotein E (APOE) genotype test were optional. The detailed information is shown in Table 1 and Additional file 1: eAppendixes 2 and 3.

**Table 1:** Demographic and clinical information collection in this multicenter study

Items	Subitems	Other information
Demographic data	Sex	Men or women
	Age at last visit	The age at patients’ last visit at each center
	Educational years	
	Onset age	The onset ages were recorded according to patients’ and/or caregivers’ chief complaints. We conducted the onset ages of cognitive impairment (*n* = 1038) and parkinsonism (*n* = 781) in this study
	Interval between cognitive impairment and parkinsonism	The absolute value of onset age of cognitive impairment minus onset age of parkinsonism among the target patients. There were 781 pieces of data were analyzed, since a total of 781 patients (418 men and 363 women; 19 patients in MCI-LB, 331 patients in DLB, 118 patients in PD-MCI and 313 patients in PDD) had parkinsonism in this study
	Course of disease	Age at last visit minus onset age
Sex ratio		Sex ratios mean the number of men divided women (men/women)
Clinical core features	Fluctuating cognition	The presence was diagnosed with three or more “yes” responses required for structured questions from caregivers confirmed by the Mayo Fluctuations Composite Scale
	Visual hallucinations	The hallucinations item of 12-item NPI was used to determine the presence of hallucination, as complaining about by the patient and/or caregiver with specifically formed and detailed VH and illusions
	Parkinsonism	This is diagnosed by having one or more spontaneous cardinal features of parkinsonism included bradykinesia, rest tremor or rigidity evaluated by the motor section (Part III) of the Movement Disorders Society Unified Parkinson’s Disease Rating Scale (MDS-UPDRS)
	RBD	It can be confirmed by caregivers who mentioned five or more behaviors that are mentioned in the RBD screening questionnaire (RBD-SQ); or this patient was diagnosed by an overnight video polysomnography
Cognitive status	MCI or dementia	
Clinical diagnosis at last visit	Probable MCI-LB, DLB, PD-MCI or PDD	The diagnostic criteria and clinical core features assessments are detailed described below
Neuropsychological assessments	Mini-Mental State Examination (Chinese version)	Scores range from 0 (severe impairment) to 30 (no impairment). It is used to evaluate global cognitive function
	Montreal Cognitive Assessment	Scores range from 0 (severe impairment) to 30 (no impairment), It is used to evaluate global cognitive function
	The Activities of daily living	Scores range from 20 (no impairment) to 80 (severe dysfunction), it is used to evaluate the functional status
	The Clinical Dementia Rating	Scores range from 0.5 (MCI), 1.0 (mild), 2.0 (moderate) to 3.0 (severe), it is used to evaluate the severity of dementia
	Neuropsychiatric Inventory*	Each subscale ranges between 0 (NPS) and 12 and the total composite score between 0 (no NPS) and 144, it is used to evaluate the presence and severity of NPS A total of 451 patients (61 patients with MCI-LB, 339 with DLB, 15 with PD-MCI and 36 with PDD) underwent NPI assessment in this study
MRI visual rating scales	Medial Temporal lobe Atrophy*	Scores range from 0 (no atrophy) to 4 (severe volume loss of hippocampal volume, it is used to evaluate the visual regional brain atrophy in the hippocampus, parahippocampal gyrus, entorhinal cortex and the surrounding cerebrospinal fluid spaces Multiplanar oblique coronal (perpendicular to the axis of the hippocampus), transverse and coronal position reconstructions were made of 3D T1-weighted images for diagnostic multisequence MRI. All of the MRI readings were reviewed by two experienced neuroradiologists double-blindly, and the final rating scores were averaged A total of 922 patients (74 patients with MCI-LB, 480 with DLB, 107 with PD-MCI and 261 with PDD) completed MRI visual rating scales
	Fazekas scales*	Scores range from 0 (no or single punctate lesion) to 3 (large confluent lesions), it is used to reflect the whole white matter lesion. The numbers of participants, the principles of MRI parameters and review are the same as described above
APOE genotype*		Genomic DNA was extracted from peripheral blood stored at -80 ℃, and the APOE gene was amplified by polymerase chain reaction. All genotypes were determined without knowledge of the patient status A total of 167 patients (40 patients with MCI-LB, 111 with DLB, 0 with PD-MCI and 16 with PDD) had the APOE genotype test

^*^It means the items or subitems were optional to provide

*RBD* rapid eye movement sleep behavior disorder, *MCI* mild cognitive impairment, *MCI-LB* mild cognitive impairment with Lewy bodies, *DLB* dementia with Lewy bodies, *PD-MCI* Parkinson’s disease with mild cognitive impairment, *PDD* Parkinson’s disease dementia, *NPS* neuropsychiatric symptoms, *MRI* magnetic resonance imaging, *APOE* apolipoprotein E

The Ethics Committees of the 22 centers approved all research activities in this multicenter study and waived informed consent because the data were pseudonymized from registers. The procedures were performed in accordance with the ethical standards of the Committee on Human Experimentation.

### Diagnostic criteria

Probable DLB: diagnosed with two or more core symptoms with or without indicative biomarkers, or only one core symptom with one or more indicative biomarkers, according to the criteria of McKeith et al. [1].

Probable MCI-LB: diagnosed if a patient had two or more core clinical features of DLB with or without the presence of a proposed biomarker (positive FP-CIT SPECT or dopamine transporter PET, and/or meta-iodobenzylguanidine scan, and/or polysomnographic confirmation of REM sleep without atonia) or only one core clinical feature plus one or more proposed biomarkers [25]. Since the consensus of the criteria for MCI-LB were in development at the time of first diagnosis, so probable MCI-LB was initially defined with a combination of MCI criteria using Petersen’s criteria in 2011 [26] and DLB criteria by McKeith in 2017 [1], with a MMSE ≥ 20 and CDR score of ≥ 0.5 [27]. The final diagnosis of probable MCI-LB was confirmed by two experienced neurologists double-blindly.

Probable PDD: diagnosed according to the clinical criteria for probable PDD, developed by the Movement Disorder Society in 2007 [28].

Probable PD-MCI: diagnosed by the diagnostic criteria developed by the Movement Disorder Society Task Force level I or level II diagnosis [29].

All patients with cognitive impairment in Lewy body disease mentioned in this study had a probable diagnosis. According to the international consensus of the “one-year rule”, DLB should be diagnosed when cognitive impairment precedes parkinsonism or begins within a year of parkinsonism, and PDD should be diagnosed when parkinsonism precedes cognitive impairment by more than one year.

### Statistical analyses

Descriptive analyses were conducted by number (proportion, %) for qualitative variables and mean [± standard deviation (SD)] or median (interquartile range) after normality tests for quantitative variables. The sex ratios were calculated by men (number)/women (number). Variables associated with diagnosis (MCI-LB, DLB, PD-MCI, and PDD) were tested using analysis of variance for quantitative variables and Chi-squared tests for qualitative variables. For comparisons of groups, Student’s *t*-test was used for normally distributed data and a Mann–Whitney *U*-test for nonparametric data, qualitative variables were assessed using a Chi-squared test. The R × C contingency tables were used for the comparison of rates among the four groups according to the diagnoses (MCI-LB, DLB, PD-MCI, and PDD), and Fisher's exact test (R × C) was used for samples with theoretical frequencies less than one in Table 3. The *P*’-values were corrected by Bonferroni correction.

Linear and logistic regression analyses were conducted to evaluate sex differences in demographic and clinical outcome measures, and represent the data with *β* ± standard error (SE). Firstly, we used two linear regression models with sex as the factor, and with demographics (age at cognitive impairment, age at parkinsonism, interval between cognitive impairment and parkinsonism, and course of disease, MRI visual scales [MTA scores (both in left and right) and Fazekas scales], and clinical assessments (C-MMSE, MoCA, ADL, CDR, and NPI and its subitems) as main outcome measures. Then logistic regressions were used to explore the relationship between four clinical core features as the dependent variables and sex ratio with other possible explicative variables in Model 2 [education, cardiometabolic conditions (hypertension, type 2 diabetes mellitus (T2DM), heart disease, and stroke), smoking and alcohol consumption, and age at last visit and course of disease]. The reference modality was “men.”

The IBM SPSS for Windows (version 25.0; IBM Corporation, Armonk, NY, USA) was used for statistical analyses, with *p* < 0.05 considered significant at the two-tailed *α* level.

## Results

### Demographic and clinical characteristics

According to our selection criteria, we assembled four groups in this study: MCI-LB (*n* = 74, mean age at last visit = 70.46 ± 7.30), DLB (*n* = 533, mean age at last visit = 72.05 ± 8.22), PD-MCI (*n* = 118, mean age at last visit = 64.69 ± 8.26), and PDD (*n* = 313, mean age at last visit = 68.06 ± 7.88). The demographic and clinical characteristics of the patients are shown in Table 2. Men had longer course of disease (*p* = 0.000), higher proportions of stroke (*p* = 0.009), smoking (*p* = 0.000) and alcohol consumption (*p* = 0.000) than women. Men performed better in C-MMSE (*p* = 0.006) and MoCA (*p* = 0.009) and had less depression (*p* = 0.000). We did not find any sex difference in the demographic information, APOE ε4 status, MTA and Fazekas scores, and ADL, CDR, and NPI scores. The sex-specific characteristics of the four groups are displayed in Additional file 1: Table S1. Women were younger at last visit (*p* = 0.04), had a shorter course of disease (*p* = 0.000), and lower scores of MTA (*p* = 0.001 in left, *p* = 0.009 in right) than men in PDD cases.

**Table 2:** Demographic and clinical characteristics of participants

Characteristics ^a^	All	Men	Women	t/Z/χ2	p-value
	*n* = 1038	*n* = 514	*n* = 524		
Age at last visit, mean (SD), y	69.89 ± 8.45	70.18 ± 8.26	69.61 ± 8.63	− 1.26	0.21
Age at CI, mean (SD), y	67.78 ± 8.14	68.17 ± 7.92	67.39 ± 8.34	− 1.63	0.10
Age at PARK^b^, mean (SD), y	66.78 ± 8.14	66.83 ± 9.04	66.16 ± 9.57	− 0.13	0.26
Interval between CI and PARK^b^, mean (SD), y	2.95 ± 2.56	2.96 ± 2.61	2.93 ± 2.50	− 0.15	0.88
Education, mean (SD), y	8.90 ± 4.54	10.09 ± 4.00	7.73 ± 4.74	− 1.17	0.24
Course of disease, mean (SD), y	3.05 ± 2.26	3.15 ± 2.40	2.95 ± 2.12	− 7.50	**0.000**
Cardiometabolic conditions^c^					
Hypertension	275 (32.54%)	127 (31.28%)	148 (33.71%)	0.57	0.45
T2DM	100 (11.83%)	50 (12.32%)	50 (11.39%)	0.17	0.68
Heart disease	116 (13.73%)	52 (12.81%)	64 (14.58%)	0.56	0.46
Stroke	109 (12.90%)	65 (16.01%)	44 (10.02%)	6.73	**0.009**
Smoking^c^	139 (16.45%)	118 (29.06%)	21 (4.78%)	90.48	**0.000**
Alcohol consumption^c^	109 (12.90%)	97 (23.89%)	12 (2.73%)	84.04	**0.000**
APOE ɛ4 carriers^d^	56 (33.53%)	17 (30.91%)	39 (34.82%)	0.25	0.62
MTA scores, mean (SD)^e^					
Left	1.19 ± 0.73	1.20 ± 0.77	1.18 ± 0.70	− 0.03	0.98
Right	1.18 ± 0.75	1.19 ± 0.79	1.18 ± 0.71	− 0.19	0.85
Fazekas scales, mean (SD)^e^	1.17 ± 0.69	1.16 ± 0.71	1.18 ± 0.68	− 0.57	0.57
C-MMSE, mean (SD)	17.86 ± 6.88	18.37 ± 7.07	17.36 ± 6.65	− 2.77	**0.006**
MoCA, mean (SD)	12.89 ± 6.51	13.44 ± 6.62	12.34 ± 6.35	− 2.76	**0.006**
ADL, mean (SD)	30.91 ± 12.86	30.47 ± 12.69	31.35 ± 13.02	− 1.66	0.10
CDR, mean (SD)	1.57 ± 0.83	1.53 ± 0.84	1.60 ± 0.83	− 1.40	0.16
NPI scores, mean (SD)^f^	12.93 ± 13.01	11.73 ± 12.44	13.93 ± 13.42	− 1.91	0.05
Delusions	161 (35.94%)	71 (34.98%)	90 (36.73%)	0.15	0.67
Hallucinations	336 (75.00%)	154 (75.86%)	182 (74.29%)	0.15	0.70
Agitation	117 (26.12%)	51 (25.12%)	66 (26.94%)	0.19	0.66
Depression	182 (40.63%)	64 (31.53%)	118 (48.15%)	12.74	**0.000**
Anxiety	161 (35.94%)	70 (34.48%)	91 (37.14%)	0.34	0.56
Euphoria	33 (7.37%)	17 (8.37%)	16 (6.53%)	0.55	0.46
Apathy	172 (38.39%)	78 (38.42%)	94 (38.37%)	0.00	0.99
Disinhibition	53 (11.83%)	23 (11.33%)	30 (12.24%)	0.09	0.77
Irritability	154 (34.38%)	75 (36.95%)	79 (32.24%)	1.09	0.30
Aberrant motor behavior	134 (29.91%)	58 (28.57%)	76 (31.02%)	0.32	0.57
Night-time behavior disturbances	321 (71.65%)	153 (75.37%)	168 (68.57%)	2.53	0.11
Appetite and eating abnormalities	112 (25.00%)	47 (23.15%)	65 (26.53%)	0.68	0.41

Bold means that the significant *P* values

*P*-value means the comparison between men and women by Mann–Whitney *U* test or *χ*^2^ test

*CI* cognitive impairment, *PARK* parkinsonism, *SD* standard deviation, *ML* memory loss, *MDs* movement disorders, *T2DM* type 2 diabetes mellitus, *APOE* apolipoprotein E, *MTA* medial temporal lobe atrophy, *C-MMSE* the Mini-Mental State Examination (Chinese version), *MoCA* the Montreal Cognitive Assessment, *ADL* the Activity of Daily Living Scale, *CDR* the clinical dementia rating, *NPI* the Neuropsychiatric Inventory

^a^Unless otherwise indicated, data are expressed as number (%) of patients

In the statistical analysis, ^b^781 patients (418 men and 363 women) had parkinsonism and the information of interval between cognitive impairment and parkinsonism; ^c^406 men and 439 women completed cardiometabolic conditions, smoking and alcohol consumption investigation; ^d^55 men and 112 women underwent APOE genotype tests; ^e^449 men and 473 women underwent MTA and Fazekas visual evaluation; ^f^203 men and 245 women underwent NPI assessment

### Sex ratios

**Fig. 1:**
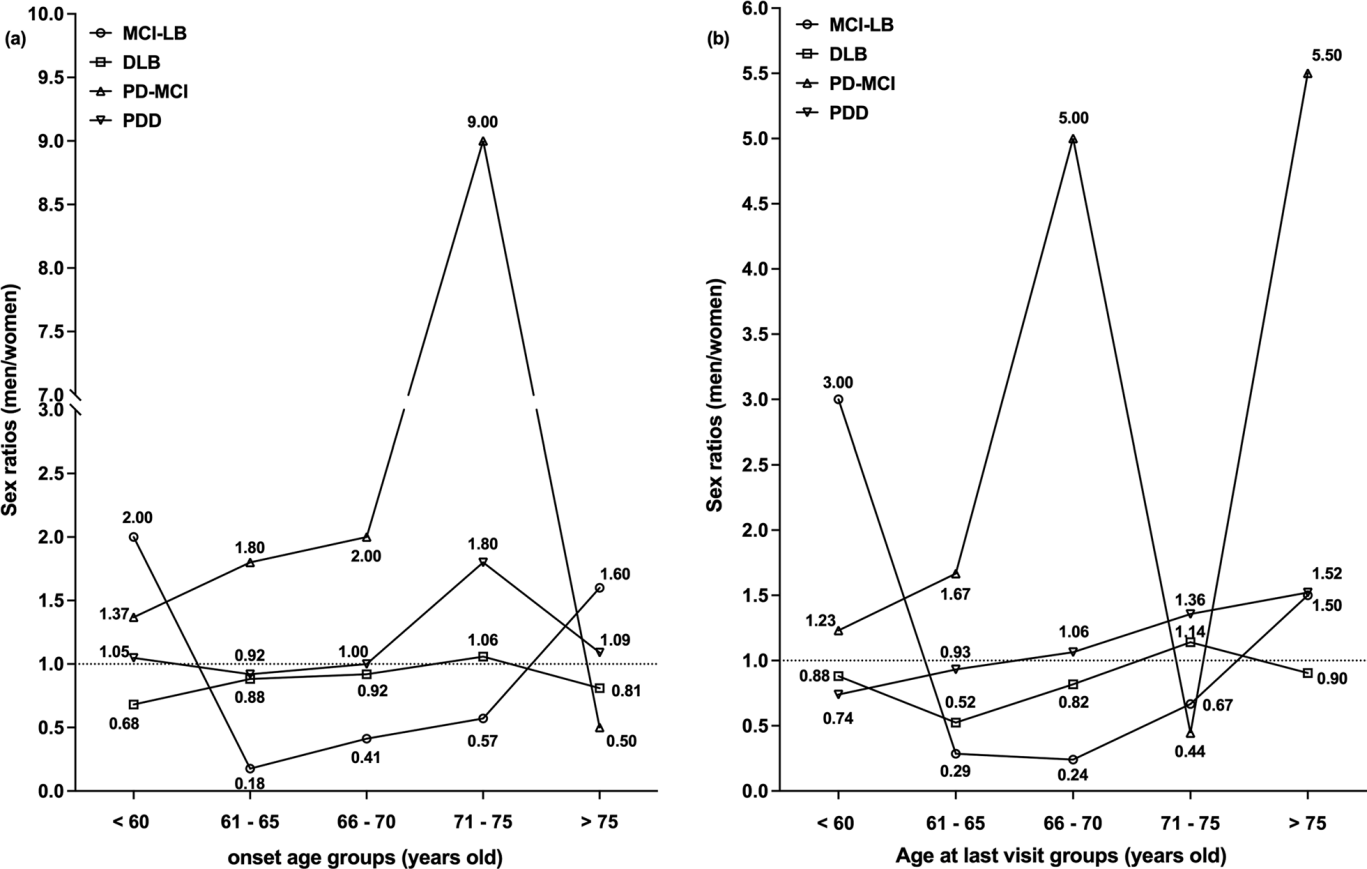
Age-specific sex ratios in patients with MCI-LB, DLB, PD-MCI and PDD. The onset age-specific (**a**) and age at last visit-specific (**b**) sex ratios are shown in this figure. And the numbers of participants in each group are described at Additional file 2. *MCI-LB* mild cognitive impairment with Lewy bodies, *DLB* dementia with Lewy bodies, *PD-MCI* Parkinson’s disease with mild cognitive impairment, *PDD* Parkinson’s disease dementia

Men comprised 35.14% (sex ratio = 0.54) for MCI-LB, 46.72% (sex ratio = 0.88) for DLB, 63.56% (sex ratio = 1.74) for PD-MCI, and 52.40% (sex ratio = 1.10) for PDD. The onset age-specific (Fig. 1a) and age at last visit-specific (Fig. 1b) sex ratios for individual diagnostic groups were shown. Women were more common among patients with MCI-LB who developed the disease between the ages of 65 and 75, whereas more men developed PD-MCI before 75 years. Sex ratios roughly increased with age, both of the ages at onset and at last visit in DLB and PDD patients. We also found sex ratios significantly differed among the four groups on the basis of education and number of core features (Table 3). A greater proportion of women were educated less than 7 years and had one or two core features. The sex ratio in favor of men increased with education in patients with MCI-LB, DLB, and PDD.

**Table 3:** The education- and number of core features-specific sex ratios in patients with MCI-LB, DLB, PD-MCI and PDD

		MCI-LB (*n* = 74)		DLB (*n* = 533)		PD-MCI (*n* = 118)		PDD (*n* = 313)		*χ*^2^	Cramer's *V*	*P*-value
		Num	Pro. (%)	Num	Pro. (%)	Num	Pro. (%)	Num	Pro. (%)			
*Education years*										80.79	0.28	0.000
0 year	Men	1	4.55	7	13.73	1	100.00	8	24.24			
	Women	21	95.45	44	86.27	0	0.00	25	75.76			
1–6 years	Men	3	15.79	55	47.83	12	70.59	34	48.57			
	Women	16	84.21	60	52.17	5	29.41	36	51.43			
≥ 7 years	Men	22	66.67	187	50.95	62	62.00	122	58.10			
	Women	11	33.33	180	49.05	38	38.00	88	41.90			
*Num. of core features*										42.14	0.20	**0.000**
One	Men	10	30.30	24	47.06	9	56.25	14	58.33			
	Women	23	69.70	27	52.94	7	43.75	10	41.67			
Two	Men	9	31.03	97	43.11	50	63.29	54	68.35			
	Women	20	68.97	128	56.89	29	36.71	25	31.65			
Three	Men	7	58.33	81	49.69	14	66.67	80	42.55			
	Women	5	41.67	82	50.31	7	33.33	108	57.45			
Four	Men	0	0.00	47	50.00	2	100.00	16	72.73			
	Women	0	0.00	47	50.00	0	0.00	6	27.27			

Bold means that the significant *P* values

*MCI-LB* mild cognitive impairment with Lewy bodies, *DLB* dementia with Lewy bodies, *PD-MCI* Parkinson’s disease with mild cognitive impairment, *PDD* Parkinson’s disease dementia, *Num.* number of patients, *Pro.* proportion

**Fig. 2:**
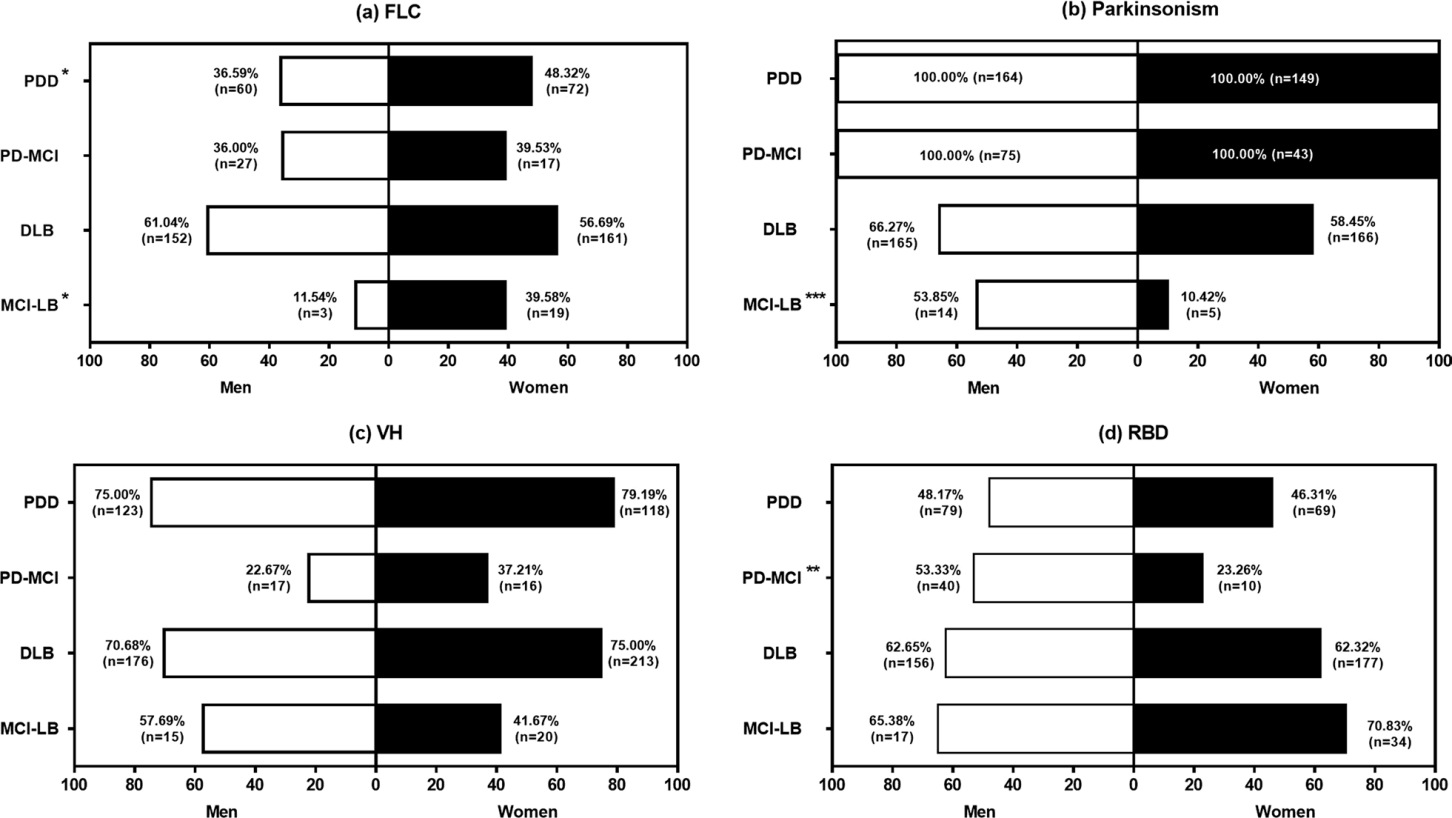
Sex ratios for core clinical features of patients with MCI-LB, DLB, PD-MCI and PDD. Figures show the proportions of FLC (**a**), parkinsonism (**b**), VH (**c**), and RBD (**d**) in women and men patients in four groups. *MCI-LB* mild cognitive impairment with Lewy bodies; *DLB* dementia with Lewy bodies, PD-MCI, Parkinson’s disease with mild cognitive impairment, *PDD* Parkinson’s disease dementia, *FLC* fluctuating cognition, *RBD* rapid eye movement sleep behavior disorder, *VH* visual hallucinations. *p < 0.05, ** p < 0.01, *** *p* < 0.001

Sex differences were also found in core clinical features of the four groups (Fig. 2). Compared with women, men with MCI-LB had more parkinsonism (53.85% vs 10.42%, *p* = 0.000), less FLC (11.54% vs 39.58%, *p* = 0.024), and those with PD-MCI had more RBD (53.33% vs 23.26%, *p* = 0.001). Men and women with DLB or PDD had similar rates of FLC, parkinsonism, VHs, and RBD.

### Associations between sex and demographic and clinical features

We evaluated the associations between sex and demographic and clinical features in Table 4. Women with PD-MCI were younger when complaining of cognitive impairment (β ± SE =  − 2.64 ± 0.97, *p* = 0.007) and parkinsonism (− 2.60 ± 1.05, *p* = 0.01) than men after adjusting education and cardiometabolic conditions; however, we found no such sex difference of age for DLB, or PDD groups. Women with PD-MCI were more likely to have lower score of C-MMSE (− 1.24 ± 0.58, *p* = 0.04), more irritability (0.95 ± 0.46, *p* = 0.04) and FLC (− 3.41 ± 1.31, *p* = 0.01), and less parkinsonism (− 2.10 ± 0.97, *p* = 0.03) than men after adjusting for age at last visit, education, course of disease, and cardiometabolic conditions in Model 2. Being female independently increased the risks of higher NPI score (*β* ± SE = 6.04 ± 1.72, *p* = 0.001), especially delusions (0.87 ± 0.34, *p* = 0.01), hallucinations (0.83 ± 0.36, *p* = 0.02), depression (1.10 ± 0.31, *p* = 0.000), and anxiety (0.80 ± 0.27, *p* = 0.003) in DLB. Moreover, being female was related to higher scores of MTA (both left, *p* = 0.03 and right, *p* = 0.05) and Fazekas (*p* = 0.04) in PD-MCI, but lower scores of MTA (both left, *p* = 0.003 and right, *p* = 0.02) in PDD.

**Table 4:** Regression models of sex, demographic and clinical features

Characteristics	MCI-LB		DLB		PD-MCI		PDD	
	Model 1	Model 2	Model 1	Model 2	Model 1	Model 2	Model 1	Model 2
Women vs. men (Ref.)								
*Demographics*^a^								
Age at CI, y	− 1.49 ± 2.40	–	− 1.05 ± 0.87	–	− 1.47 ± 1.75	–	− 2.64 ± 0.97**	–
Age at PARK, y	− 28.02 ± 4.07***	–	− 0.46 ± 1.03	–	− 1.98 ± 1.92	–	− 2.60 ± 1.05*	–
Interval between CI and PARK, y	2.72 ± 2.76	–	0.54 ± 0.25*	–	0.49 ± 0.67	–	− 0.02 ± 0.42	–
Course of disease, y	0.05 ± 0.78	–	0.34 ± 0.20	–	0.28 ± 0.55	–	− 0.53 ± 0.35	–
*MRI visual scales*^a^								
MTA scores								
Left	–	− 0.09 ± 0.18	–	− 0.02 ± 0.07	–	0.19 ± 0.09*	–	− 0.20 ± 0.07**
Right	–	− 0.01 ± 0.18	–	− 0.02 ± 0.08	–	0.18 ± 0.09*	–	− 0.16 ± 0.07*
Fazekas scales	–	0.14 ± 0.26	–	− 0.01 ± 0.07	–	0.23 ± 0.11*	–	− 0.08 ± 0.07
*Clinical assessments*^a^								
C-MMSE	–	− 1.24 ± 0.58*	–	0.45 ± 0.67	–	0.05 ± 0.28	–	− 0.02 ± 0.74
MoCA	–	− 1.01 ± 0.54	–	0.28 ± 0.55	–	0.61 ± 0.47	–	− 0.03 ± 0.67
ADL	–	–	–	–	–	0.95 ± 1.51	–	− 0.30 ± 1.53
CDR	–	–	–	–	–	− 0.08 ± 0.08	–	− 0.02 ± 0.09
NPI	–	3.63 ± 2.04	–	6.04 ± 1.72**	–	–	–	− 1.82 ± 4.46
Delusions	–	0.45 ± 0.26	–	0.87 ± 0.34*	–	–	–	0.12 ± 0.60
Hallucinations	–	0.61 ± 0.44	–	0.83 ± 0.36*	–	–	–	0.43 ± 1.01
Agitation	–	0.33 ± 0.43	–	0.17 ± 0.22	–	–	–	− 0.004 ± 0.13
Depression	–	0.26 ± 0.35	–	1.10 ± 0.31***	–	–	–	0.02 ± 0.03
Anxiety	–	0.28 ± 0.20	–	0.80 ± 0.27**	–	–	–	− 1.01 ± 0.66
Euphoria	–	0.07 ± 0.13	–	− 0.15 ± 0.10	–	–	–	− 0.03 ± 0.06
Apathy	–	− 0.29 ± 0.31	–	0.49 ± 0.33	–	–	–	− 0.86 ± 1.30
Disinhibition	–	0.31 ± 0.23	–	− 0.02 ± 0.13	–	–	–	0.13 ± 0.10
Irritability	–	0.95 ± 0.46*	–	0.25 ± 0.24	–	–	–	− 0.42 ± 0.48
Aberrant motor behavior	–	0.33 ± 0.29	–	0.91 ± 0.34**	–	–	–	0.63 ± 0.60
Night-time behavior disturbances	–	0.002 ± 0.37	–	0.59 ± 0.36	–	–	–	0.13 ± 1.22
Appetite and eating abnormalities	–	0.33 ± 0.31	–	0.27 ± 0.24	–	–	–	− 0.71 ± 0.50
*Core clinical features*^b^								
RBD	–	− 0.05 ± 1.11	–	0.23 ± 0.24	–	− 1.85 ± 0.53**	–	− 0.28 ± 0.29
Parkinsonism	–	− 2.10 ± 0.97*	–	− 0.61 ± 0.25*	–	–	–	–
Fluctuating cognition	–	3.41 ± 1.31**	–	− 0.26 ± 0.23	–	0.70 ± 0.50	–	0.55 ± 0.30
Visual hallucinations	–	− 1.01 ± 0.99	–	0.08 ± 0.27	–	0.33 ± 0.49	–	0.50 ± 0.38

Bold means that the significant *P* values

In the statistical analysis of Model 1 and Model 2, 62 patients with MCI-LB, 437 patients with DLB, 104 patients with PD-MCI, and 242 patients with PDD were analyzed for the age at CI, course of disease, C-MMSE, MOCA, ADL, and CDR, as well as four core clinical features; 17 patients with MCI-LB, 292 patients with DLB, 104 patients with PD-MCI, and 242 patients with PDD were analyzed for the age at PARK and the interval between CI and PARK; 62 patients with MCI-LB, 434 patients with DLB, 102 patients with PD-MCI, and 236 patients with PDD were analyzed for MRI visual scales; and 61 patients with MCI-LB, 339 patients with DLB, 12 patients with PD-MCI, and 36 patients with PDD were analyzed for NPI. Data represent β ± standard error by linear regressions (a) or logistic regressions (b). *p < 0.05, ** p < 0.01, *** *p* < 0.001

Model 1: with correction for education, cardiometabolic conditions (hypertension, type 2 diabetes mellitus, heart disease, stroke), smoking and alcohol consumption by linear regressions

Model 2: Model 1 with correction for age at last visit and course of disease

*MCI-LB* mild cognitive impairment with Lewy bodies, *DLB* dementia with Lewy bodies, *PD-MCI* Parkinson’s disease with mild cognitive impairment, *PDD* Parkinson’s disease dementia, *CI* cognitive impairment, *PARK* parkinsonism, *MRI* Magnetic Resonance Imaging, *MTA* medial temporal lobe atrophy, *C-MMSE* the Mini-Mental State Examination (Chinese version), *MoCA* the Montreal Cognitive Assessment, *ADL* the Activity of Daily Living Scale, *CDR* the clinical dementia rating, *NPI* the Neuropsychiatric Inventory, *RBD* rapid eye movement sleep behavior disorder

## Discussion

In the present study, DLB and MCI-LB were more prevalent in women than men, while PD-MCI and PDD were more prevalent in men than women. Women had more frequent depression, shorter course of disease, and lower C-MMSE and MoCA scores than men. In addition, being female was associated with severe neuropsychiatric symptoms (NPS) in DLB, and lower MTA and Fazekas scores in PDD. This multicenter study first described the sex-specific characteristics of LBD in the prodromal and dementia stages, reflecting the clinical practice of LBD in China to a certain extent.

### Sex ratios in cognitive impairment in Lewy body disease

We found that women were more common in DLB (64.86%), while men were slightly predominant in PDD with a proportion of 52.40%, which tended to differ for Western populations. A retrospective cohort in UK aiming to evaluate mortality of DLB showed a slight women predominance (51.4%) [10]. Mouton et al. [3] conducted a cross-sectional clinical study with 10 309 DLB and showed women comprising 54.7% for DLB. Traditionally, scientific literature on sex distribution in dementia has reported a more pronounced or roughly equal prevalence of men in DLB and PDD [2]. Mouton et al. also demonstrated a predominance of men (54.6%) in PDD, in line with previous literature reporting a more pronounced prevalence of men in PDD (in Sweden, *n* = 297, 61.3% were men [5]; in China, *n* = 107, 57.9% were men [8]). We firstly reported the sex ratios in MCI-LB and PD-MCI, and found a strong women predominance with 35.14% (sex ratio = 0.54) for MCI-LB, and a strong male predominance with 63.56% (sex ratio = 1.74) for PD-MCI. In addition, the sex ratios varied in different groups classified by age and education years. With the increase of age, in terms of the age at last visit or at onset, sex ratios in favor of men generally showed a rising trend in patients with MCI-LB, DLB, PD-MCI and PDD. PD was significantly prevalent in men, thus it was easy to understand that PD-MCI and PDD, as cognitive disorders progressing from PD, have a higher proportion in men than in women. Women accounted for the majority of DLB patients between 60 and 75 years of age, although the sex ratio gradually decreased with age. However, the sex ratio in favor of females increased with age for DLB patients older than 75 years old in Mouton et al*.* 's research, as in our study. We supposed that women might be more likely to seek help for dementia in China [30], or have more neuropsychiatric symptoms [31] that are noted during the course of the disease, which led to women predominance. Moreover, these difference may be due to either one or a combination of study design, sample-size, as well as potential ethnoracial, genetic, environmental and occupational factors. We also found that the proportion of men gradually rose both in DLB and PDD with increased education level. This finding may be due to the educational imbalance between older Chinese men and women [32], in that men were educated longer than women.

DLB is diagnosed by clinical symptoms and biomarkers according to current criteria, and the “1-year rule” is remaining supported to distinguish DLB from PDD in clinical practice. Cases of suspected DLB presenting with dementia alone, that is without parkinsonism, may also have been missed and classified as AD, particularly if no other core feature was reported in the patient’s records. Moreover, for those DLB patients with neuropsychiatric symptoms, more “mental disorders” will be diagnosed and treated in the department of psychiatry. Neuropathological studies reported that men were more likely to have pure neocortical (“diffuse”) or intermediate (“limbic”) Lewy body pathologies, whereas women had more AD pathology and cerebrovascular disease [7, 33], and thus more women would also be classified as AD. There are reasons to believe that the clinically suspected DLB is underestimated, particular in women, and women might account for a larger proportion of DLB.

### Sex difference in clinical features

Formal studies demonstrated a significant but controversial association between clinical symptoms and sex in DLB, but associations have not been extensively studied for PDD, MCI-LB, or PD-MCI. In this clinical multicenter cohort, women had a non-significantly higher proportion of VHs, and no sex differences were found in other core clinical features. Chiu et al*.* [11] showed that VHs were more common in women with clinical DLB adjusted for age and disease severity, with the same finding in the cohort of van de Beek [34]. In a Japanese study with 234 clinical DLB patients [35], VHs and FLC were non-significantly more prevalent for women, while parkinsonism (*p* = 0.027) and RBD (*p* = 0.000) were more prevalent for men. In a pathological DLB study [36] based on the NACC Neuropathology Data Set, fewer women had VHs (*p* = 0.009), RBD (*p* = 0.007), or parkinsonism (*p* = 0.007) compared with men. In patients with MCI-LB, we found that RBD was the most common but with no sex difference; women had more FLC (39.58% vs 11.54%, *p* = 0.024) and men had more parkinsonism (53.85% vs 10.42%, *p* = 0.000). We also found that RBD was the second most common clinical feature after parkinsonism and was more frequent for men (53.33% vs 23.26%, *p* = 0.001) with PD-MCI. In the prodromal and dementia stages of LBD, patients show different sex predominance in core clinical features. Previous studies in patients with PD and PDD showed that RBD was more common in men [12, 37], and its presence and severity were associated with decreased cerebrospinal fluid (CSF) alpha-synuclein level [38, 39]. Although Yu et al. [39] showed that omen had lower CSF alpha-synuclein levels (1429 ± 164 vs 1831 ± 60, *p* = 0.02) than men, and only one longitudinal study [40] revealed a significant correlation between estimated changes in alpha-synuclein level and RBD-SQ scores (*p* = 0.001, data not available), we still do not know how sex influences occurrence of RBD by influencing alpha-synuclein levels. The FLCs included memory, attention, executive functions, language, and visuospatial function fluctuation during the day and over weeks. Women with MCI-LB had worse cognitive function in the current study, possibly contributing to the women’s predominance of FLC. The frequency of FLC in MCI-LB cases has not been determined.

Consistent with previous literature [12, 41], NPS occurred frequently and severely in DLB female cases in our cohort. Several studies also showed that, even in the earliest disease stages, NPS can be present [27, 42, 43], but we found no significant sex differences in patients with MCI-LB. The PDD patients showed significant sex differences with more hippocampal and white-matter damage in men, possibly because 17β-estradiol (E2) conveys neuroprotective effects on the hippocampal and cardio-cerebral vascular system in women [44, 45]. Previous studies have demonstrated that estradiol levels have effect on cognition and memory during menopause transition, and women could have better cognitive and memory performance in relevant tasks after estradiol-based hormone therapy (E2-HT) [46–48]. According to the baseline information of Cardiovascular Risk factors, Aging and Dementia cohort [47], women who had used E2-HT for > 5 years had better scores in global cognition, episodic memory, and psychomotor speed tests at baseline than women who had used E2-HT for less than five years or non-users. A prospective, randomized, double-blind, placebo-controlled trial in Korea [48] showed that, when comparing with the control group, menopausal hormone therapy using percutaneous E2 gel and micronized progesterone could significantly reduce the deterioration of MoCA score, and increase the scores of MMSE and MoCA at 24 months for women with MCI. Confusingly, women were associated with higher scores of MTA (both left and right) and Fazekas in PD-MCI, but lower scores of MTA (both left and right) in PDD in linear regression models after adjusting for demographic and clinical features. This may be due to “phased” estradiol protection mechanisms, meaning that premenopausal women maintain high levels of estradiol to protect the hippocampal and cardio-cerebral vascular system, but for a period after menopause (“MCI window”), women show a sudden drop of estradiol levels, a period of time when women have more severe brain damage. However, with the increase of age and entering another stage after menopause (“dementia window”), this kind of damage tends to be gentle in elderly women. The authors consider this hypothesis bold and interesting, and a mechanism worth exploring.

### Strength and limitations

The main strength of this study is that this is the first multicenter study in China utilizing a large sample size and focuses on the prodromal stage of LBD. These findings reflect clinical facts, also can represent the clinical characteristics of cognitive impairment in Lewy body disease in Eastern populations and enrich the literature that predominantly compiled of patients from European descent. The clinical diagnosis was made by a physician experienced in neurodegenerative disease, largely supported using PET–CT and CSF biomarkers, as well as regular follow-up to improve diagnostic accuracy. All of the clinical information was reviewed from medical records, which reduced recall bias.

Uncertainty may also occur in deciding how patients exhibiting both MCI/dementia and parkinsonism are best categorized. There are few comparative studies on MCI-LB and PD-MCI, and having no definitive differential diagnostic criteria for them. Thus, we are still referring to the “one-year rule”, similar to that used to separate DLB and PDD, to distinguish some clinical MCI-LB and PD-MCI cases if the onset and order of parkinsonism and cognitive impairment can be clearly established [25]. There were significant neuropathological differences between DLB and PDD, as DLB had greater severity of CAA than PDD [49] and showed higher seeding activity of disease-associated alpha-synuclein than PD [50]. Nevertheless, the diagnosis was based on clinical findings rather than postmortem finding, which might cause diagnostic bias in this study. Additionally, the lack of Unified Parkinson Disease Rating Scale records further affected our research on motor symptoms in cognitive impairment in Lewy body disease. Insufficient accumulation of PET–CT and CSF biomarkers in each center, and incomplete information also affected our further analysis. Finally, the cohort was all Chinese patients in memory clinics but not a community cohort, and had low number of subjects in MCI stage, which might lead to the sampling bias, as well as possible differences with other regions and so limited the generalizability.

### Perspectives and significance

This multicenter clinical cohort indicated sex differences in cognitive impairment in Lewy body disease, wherein women were predominant in DLB and MCI-LB cases, and men predominant in PD-MCI and PDD cases. Women seemed to have more frequent and severe NPS, and poorer cognition in DLB. These findings reinforce the arguments that DLB is a distinct disease from PDD, and is not the same disease even in the prodromal stage, thus it should not be confused. More importantly, due to the sex differences in clinical symptoms, it is essential for adopting sex-sensitive strategies for management of dementia. Further research to explore the role of sex differences of in the pathogenesis of LBD may contribute to the sex-specific treatment of dementia.

## Supplementary Information

**Additional file 1: eAppendix 1. **Information of participating clinics**. eAppendix 2. **Details of APOE genotyping. **eAppendix 3. **MRI parameters and review. **Table S1. **Sex-specific characteristics of the four groups

**Additional file 2:** Onset age groups (years old) and Age at last visit groups (years old).

## Data Availability

The data that support the findings of this study are available from the corresponding author upon reasonable request.
