# Supplementary material for: Sex differences in clinical cognitive impairment with Lewy bodies: a Chinese multicenter study
Source: Biol Sex Differ. 2022 Oct 1;13:55. doi: 10.1186/s13293-022-00464-w (PMC9526942; doi:10.1186/s13293-022-00464-w)
Supplement: Supplementary file 1 — Additional file 1: eAppendix 1. Information of participating clinics. eAppendix 2. Details of APOE genotyping. eAppendix 3. MRI parameters and review. Table S1. Sex-specific characteristics of the four groups [file 13293_2022_464_MOESM1_ESM.docx]

**Sex differences in clinical cognitive impairment with Lewy bodies: a Chinese multicenter study**

Jinghuan Gan ^1^, Zhichao Chen ^2^, Zhihong Shi ^3^, Xudong Li ^1^, Shuai Liu ^3^, Yiming Liu ^4^, Hongcan Zhu ^5^, Lu Shen ^6^, Guili Zhang ^1^, Yong You ^7^, Qihao Guo ^8^, Nan Zhang ^9^, Yang Lv ^10^, Baozhi Gang ^11^, Junliang Yuan ^12^, and Yong Ji ^1,3^*

^1^ Department of Neurology, Beijing Tiantan Hospital, Capital Medical University, China National Clinical Research Center for Neurological Diseases, Beijing, China;

^2^ Department of Neurology, Beijing Friendship Hospital, Capital Medical University, Beijing, China;

^3^ Tianjin Key Laboratory of Cerebrovascular and of neurodegenerative diseases, Tianjin dementia institute; Department of Neurology, Tianjin Huanhu Hospital, Tianjin, China;

^4^ Department of Neurology, Qilu hospital, Shandong University, Shandong, China;

^5^ Department of Neurology, The First Affiliated Hospital of Zhengzhou University, Zhengzhou, Henan, China;

^6^ Department of Neurology, Xiangya Hospital, Central South University, Hunan, China;

^7^ Department of Neurology, Second Affiliated Hospital of Hainan Medical University, Haikou, China;

^8^ Department of Gerontology, Shanghai Jiao Tong University Affiliated Sixth People's Hospital, Shanghai, China;

^9^ Department of Neurology, Tianjin Medical University General Hospital, Tianjin, China;

^10^ Department of Geriatrics, the First Affiliated Hospital of Chongqing Medical University, Chongqing, China;

^11^ Department of Neurology, The First Affiliated Hospital of Harbin Medical University, Harbin, China;

^12^ Department of Neurology, Peking University Sixth Hospital, Beijing, China.

**Corresponding Author:**

Yong Ji, MD, PhD., (a) Department of neurology, Beijing Tiantan Hospital, Capital Medical University; China National Clinical Research Center for Neurological Diseases, No. 119 Nansihuan xilu, Fengtai District, Beijing, 100070, China; (b) Department of Neurology, Tianjin Huanhu Hospital, Tianjin Key Laboratory of Cerebrovascular and of Neurodegenerative Diseases, Tianjin Dementia Institute, 6 Jizhao Road, Jinnan District, Tianjin, 300350, China. Tel: 86+13612048681; E-mail: jiyong@ccmu.edu.cn

**eAppendix 1** Information of participating clinics

The 22 participating memory clinics of China Lewy Body Disease Collaborative Alliance include Beijing Tiantan Hospital, Tianjin Huanhu Hospital, Xiangya Hospital of Central South University, Shanghai Jiao Tong University Affiliated Sixth People's Hospital, Peking University Sixth Hospital, Beijing Hospital of the Ministry of Health, Qilu Hospital of Shandong University, Tianjin Medical University General Hospital, Zhongshan Hospital of Fudan University, The First Affiliated Hospital of Zhengzhou University, The First Affiliated Hospital of Harbin Medical University, the First Affiliated Hospital of Chongqing Medical University, Second Affiliated Hospital of Hainan Medical University, Shengjing Hospital Affiliated to China Medical University, the First Hospital of Shanxi Medical University, The First Hospital of Hebei Medical University, Research Institute of Traditional Chinese Medicine, First Teaching Hospital of Tianjin of University Traditional Chinese Medicine, Tangshan Worker Hospital, Tianjin first center hospital, The First Hospital of Changsha, and The First People’s Hospital of Shenyang. Each memory clinic was required to be a tertiary hospital, and showed interest in LBD research. All centers reside in Beijing (n = 3), Tianjin (n = 5), Shanghai (n = 2), Hunan (n = 2), Shandong (n = 1), Henan (n = 1), Harbin (n = 1), Chongqing (n = 1), Liaoning (n = 2), Hainan (n = 1), Shanxi (n = 1) and Hebei (n = 2) provinces.

**eAppendix 2** Details of APOE genotyping

Genomic DNA was extracted from peripheral blood stored at -80 ℃, and the APOE gene was amplified by polymerase chain reaction (PCR). The PCR primers were: 5′-AGGAACAACTGACCCCGGT-3′ (upstream) and 5′-TGCTCCTTCACCTCGTCCA-3′ (downstream). Each amplification reaction contained 1 µl DNA, 1 ul primers, 1 µl of 10% dNTP, 5 µl Taq Buffer, 5 µl 25 mM MgCl_2_, 0.5 µl Taq DNA polymerase (5 U/µl), and 35.5 µl double-distilled water. The thermal reactor was programmed as follows: initial denaturation at 94°C for 3 min, 35 cycles at 94°C for 30 s, annealing at 57°C for 35 s, extension at 72°C for 40s, and final extension at 72°C for 10 min. The amplification product (50 μl) was purified by a product purification kit (SK 1141). The purified product was sequenced by ABI 3730xl DNA analyzer. We determined all genotypes without knowledge of the patient status.

**eAppendix 3** MRI parameters and review

In this study, 922 patients (74 patients with MCI-LB, 480 patients with DLB, 107 patients with PD-MCI and 261 patients with PDD) underwent diagnostic multisequence 3.0-Tesla MRI, which included a sagittal 3D T1-weighted gradient-echo sequence (TR = 11 ms, TE = 4.94 ms, Flip angle = 15°, Image matrix = 232^*^256, Averages = 3, Concatenations = 1), a transverse T2-weighted fluid-attenuated inversion-recovery sequence (TR = 8570 ms, TE = 95 ms, Flip angle = 130°, Image matrix = 218^*^256, Averages = 1, Concatenations = 2) and a susceptibility-weighted imaging sequence (TR = 28 ms, TE = 20 ms, Flip angle = 15°, Image matrix = 221^*^320, Averages = 1, Concatenations = 1). All MRI was performed with whole-brain coverage.

Multiplanar oblique coronal (perpendicular to the axis of the hippocampus), transverse and coronal position reconstructions were made of 3D T1-weighted images. All of the MRI readings were reviewed by two experienced neuroradiologists double-blindly, and the final rating scores are averaged.

**Table S1** Sex-specific characteristics of the four groups

| **Characteristics ^a^** | **MCI-LB (1)** | | **PD-MCI (2)** | | **DLB (3)** | | **PDD (4)** | | **P-value** |
| --- | --- | --- | --- | --- | --- | --- | --- | --- | --- |
|  | **Men** | **Women** | **Men** | **Women** | **Men** | **Women** | **Men** | **Women** |  |
| **Age at last visit,**  median (IQR), y | 72.00  (66.75, 79.00) | 68.00  (66.25, 72.50) | 65.00  (61.00, 70.00) | 64.00  (58.00, 71.00) | 73.00  (67.00, 78.00) | 72.00  (66.00, 78.00) | 69.00  (63.00, 75.00) | 67.00  (62.00, 73.00) | P_4_ = 0.04 |
| **Age at CI,**  median (IQR), y | 70.00  (64.75, 78.00) | 66.00  (64.25, 70.75) | 64.00  (61.00, 70.00) | 64.00  (58.00, 70.00) | 69.00  (64.00, 75.00) | 69.00  (62.25, 75.00) | 67.00  (62.00, 75.00) | 66.00  (61.00, 71.50) | NA |
| **Age at PARK ^b^,**  median (IQR), y | 76.00  (66.75, 79.00) | 67.00  (65.50, 68.50) | 61.00  (56.00, 68.00) | 60.00  (54.00, 65.00) | 72.00  (67.00, 76.00) | 71.00  (65.00, 77.00) | 64.00  (59.00, 72.00) | 63.00  (59.00, 70.00) | NA |
| **Interval between CI**  **and PARK ^b^,**  median (IQR), y | 2.00  (1.00, 2.25) | 1.50  (1.25, 3.00) | 2.00  (1.00, 5.00) | 3.00  (1.00, 5.00) | 2.00  (1.00, 3.00) | 2.00  (1.00, 3.63) | 3.00  (1.00, 5.00) | 2.00  (1.00, 4.00) | P_3_ = 0.01; P_4_ = 0.004 |
| **Education,**  median (IQR), y | 12.00  (9.00, 16.00) | 3.00  (0.00, 6.00) | 12.00  (9.00, 14.00) | 9.00  (9.00,12.00) | 9.00  (6.50, 12.00) | 9.00  (6.00, 12.00) | 9.00  (6.00, 12.00) | 8.00  (6.00, 9.00) | P_1_, P_2_, and P_4_ = 0.000 |
| **Course of disease,**  median (IQR), y | 2.00  (1.00, 3.00) | 2.00  (1.00, 3.00) | 2.00  (1.00, 40.00) | 3.00  (1.00, 4.00) | 2.00  (2.00, 4.00) | 3.00  (2.00, 4.00) | 3.00  (1.00, 5.00) | 1.00  (1.00, 3.00) | P_4_ = 0.000 |
| **Cardiometabolic conditions ^c^** | |  |  |  |  |  |  |  |  |
| **Hypertension** | 5 (26.32%) | 22 (51.16%) | 16 (24.62%) | 10 (25.64%) | 66 (33.33%) | 69 (28.87%) | 40 (32.26%) | 47 (39.83%) | NA |
| **T2DM** | 3 (15.79%) | 6 (13.95%) | 4 (6.15%) | 3 (7.69%) | 27 (13.64%) | 27 (11.30%) | 16 (12.90%) | 14 (11.86%) | NA |
| **Heart disease** | 3 (15.79%) | 14 (32.56%) | 9 (13.85%) | 3 (7.69%) | 28 (14.14%) | 33 (13.81%) | 12 (9.68%) | 14 (11.86%) | NA |
| **Stroke** | 0 (0.00%) | 5 (11.63%) | 5 (7.69%) | 3 (7.69%) | 40 (20.20%) | 28 (11.72%) | 20 (16.13%) | 8 (6.78%) | P_3_ = 0.02; P_4_ = 0.02; |
| **Smoking ^c^** | 6 (31.58%) | 1 (2.33%) | 13 (20.00%) | 0 (0.00%) | 72 (36.36%) | 14 (5.86%) | 27 (21.77%) | 6 (5.08%) | P_1_ = 0.002; P_2_ = 0.003;  P_3_ = 0.000; P_4_ = 0.000 |
| **Alcohol consumption ^c^** | 6 (31.58%) | 1 (2.33%) | 17 (26.15%) | 3 (7.69%) | 51 (25.76%) | 4 (1.67%) | 23 (18.55%) | 4 (3.39%) | P_1_ = 0.003; P_2_ = 0.04;  P_3_ = 0.000; P_4_ = 0.000 |
| **APOE ɛ4 carriers ^d^** | 2 (50.00%) | 1 (2.78%) | NA | NA | 13 (31.71%) | 35 (50.00%) | 2 (20.00%) | 3 (50.00%) | P_1_ = 0.02 |
| **MTA scores,** median (IQR) ^e^ | |  |  |  |  |  |  |  |  |
| **Left** | 1.00  (0.00, 1.00) | 1.00  (0.00, 1.00) | 0.00  (0.00, 0.00) | 0.00  (0.00, 1.00) | 1.00  (0.00, 1.00) | 1.00  (0.00, 1.00) | 1.00  (1.00, 1.00) | 1.00  (1.00, 1.00) | P_4_ = 0.001 |
| **Right** | 1.00  (0.00, 1.00) | 1.00  (0.00, 1.00) | 0.00  (0.00, 0.00) | 0.00  (0.00, 1.00) | 1.00  (0.00, 1.00) | 1.00  (0.00, 1.00) | 1.00  (1.00, 1.00) | 1.00  (1.00, 1.00) | P_4_ = 0.009 |
| **Fazekas scales ^e^,**  median (IQR) | 1.00  (0.00, 1.00) | 1.00  (0.00, 1.00) | 0.00  (0.00, 1.00) | 0.50  (0.00, 1.00) | 1.00  (1.00, 2.00) | 1.00  (1.00, 2.00) | 1.00  (1.00, 1.00) | 1.00  (1.00, 1.00) | NA |
| **C-MMSE,**  median (IQR) | 25.50  (24.00, 27.00) | 21.00  (21.00, 24.00) | 26.00  (25.00, 27.00) | 26.00  (25.00, 27.00) | 15.00  (11.00, 20.00) | 15.00  (10.00, 18.75) | 21.00  (15.00, 23.00) | 20.00  (17.00, 23.00) | P_1_ = 0.000 |
| **MoCA,**  median (IQR) | 21.00  (20.00, 23.00) | 18.00  (18.00, 20.00) | 22.00  (21.00, 24.00) | 22.00  (21.00, 24.00) | 10.00  (6.00, 14.00) | 9.00  (5.00, 13.00) | 15.00  (11.00, 18.00) | 14.00  (10.00, 17.00) | P_1_ = 0.000 |
| **ADL,**  median (IQR) | 20.00  (20.00, 20.00) | 20.00  (20.00, 20.00) | 20.00  (20.00, 20.00) | 20.00  (20.00, 20.00) | 32.00  (24.00, 43.50) | 32.00  (25.00, 49.00) | 24.00  (22.00, 33.75) | 26.00  (22.00, 32.00) | NA |
| **CDR,**  median (IQR) | 0.50  (0.50, 0.50) | 0.50  (0.50, 0.50) | 0.50  (0.50, 0.50) | 0.50  (0.50, 0.50) | 2.00  (1.00, 2.00) | 2.00  (1.00, 3.00) | 1.00  (1.00, 2.00) | 1.00  (1.00, 2.00) | NA |
| **NPI ^f^,**  median (IQR) | 2.00  (1.00, 5.00) | 2.00  (1.00, 4.25) | 4.00  (1.75, 7.25) | Median=7.50 | 9.00  (3.25, 20.00) | 14.00  (6.00, 23.00) | 6.00  (1.00, 21.25) | 8.00  (2.00, 13.25) | P_3_ = 0.01 |
| **Delusions** | 1 (5.26%) | 7 (16.67%) | 0 (0.00%) | 0 (0.00%) | 64 (43.24%) | 81 (42.41%) | 6 (23.08%) | 2 (20.00%) | NA |
| **Hallucinations** | 13 (68.42%) | 20 (47.62%) | 4 (40.00%) | 0 (0.00%) | 121 (81.76%) | 153 (80.10%) | 16 (61.54%) | 9 (90.00%) | NA |
| **Agitation** | 3 (15.79%) | 6 (14.29%) | 1 (10.00%) | 1 (50.00%) | 44 (29.73%) | 58 (30.37%) | 3 (11.54%) | 1 (10.00%) | NA |
| **Depression** | 2 (10.53%) | 7 (16.67%) | 2 (20.00%) | 1 (50.00%) | 55 (37.16%) | 108 (56.54%) | 5 (19.23%) | 2 (20.00%) | P_3_ = 0.000 |
| **Anxiety** | 2 (10.53%) | 9 (21.43%) | 1 (10.00%) | 0 (0.00%) | 60 (40.54%) | 81 (42.41%) | 7 (26.92%) | 1 (10.00%) | NA |
| **Euphoria** | 0 (0.00%) | 3 (7.14%) | 0 (0.00%) | 0 (0.00%) | 16 (10.81%) | 13 (6.81%) | 1 (3.85%) | 0 (0.00%) | NA |
| **Apathy** | 4 (21.05%) | 7 (16.67%) | 1 (10.00%) | 1 (50.00%) | 65 (43.92%) | 82 (42.93%) | 8 (30.77%) | 4 (10.00%) | NA |
| **Disinhibition** | 2 (10.53%) | 4 (9.52%) | 0 (0.00%) | 0 (0.00%) | 20 (13.51%) | 25 (13.09%) | 1 (3.85%) | 1 (10.00%) | NA |
| **Irritability** | 3 (15.79%) | 4 (9.52%) | 0 (0.00%) | 1 (50.00%) | 66 (44.59%) | 73 (38.22%) | 6 (23.08%) | 1 (10.00%) | NA |
| **Aberrant motor**  **behavior** | 0 (0.00%) | 3 (7.14%) | 1 (10.00%) | 0 (0.00%) | 51 (34.46%) | 70 (36.65%) | 6 (23.08%) | 3 (30.00%) | NA |
| **Night-time behavior**  **disturbances** | 14 (73.68%) | 10 (23.81%) | 10 (100.00%) | 2 (100.00%) | 110 (74.32%) | 147 (76.96%) | 19 (73.08%) | 9 (90.00%) | P_1_ = 0.000 |
| **Appetite and eating**  **abnormalities** | 2 (10.53%) | 4 (9.52%) | 2 (20.00%) | 2 (100.00%) | 35 (23.65%) | 58 (30.37%) | 8 (30.77%) | 1 (10.00%) | NA |

^a^ Unless otherwise indicated, data are expressed as number (%) of patients. In the statistical analysis, ^b^ 781 patients [418 men (14 patients with MCI-LB, 165 patients with DLB, 75 patients with PD-MCI, 164 patients with PDD) and 363 women (5 patients with MCI-LB, 166 patients with DLB, 43 patients with PD-MCI, 149 patients with PDD)] had parkinsonism and the information of interval between cognitive impairment and parkinsonism; ^c^ 406 men (19 patients with MCI-LB, 198 patients with DLB, 65 patients with PD-MCI, 124 patients with PDD) and 439 women (43 patients with MCI-LB, 239 patients with DLB, 39 patients with PD-MCI, 118 patients with PDD) completed cardiometabolic conditions, smoking and alcohol consumption investigation; ^d^ 55 men (4 patients with MCI-LB, 41 patients with DLB, 0 patients with PD-MCI, 10 patients with PDD) and 112 women (36 patients with MCI-LB, 70 patients with DLB, 0 patients with PD-MCI, 6 patients with PDD) underwent APOE genotype tests; ^e^ 449 men (26 patients with MCI-LB, 219 patients with DLB, 67 patients with PD-MCI, 137 patients with PDD) and 473 women (48 patients with MCI-LB, 261 patients with DLB, 40 patients with PD-MCI, 124 patients with PDD) underwent MTA and Fazekas visual evaluation; ^f^ 203 men (19 patients with MCI-LB, 148 patients with DLB, 10 patients with PD-MCI, 26 patients with PDD) and 245 women (42 patients with MCI-LB, 191 patients with DLB, 2 patients with PD-MCI, 10 patients with PDD) underwent NPI assessment. And 514 men (26 patients with MCI-LB, 249 patients with DLB, 75 patients with PD-MCI, and 164 patients with PDD) and 524 women (48 patients with MCI-LB, 284 patients with DLB, 43 patients with PD-MCI, and 149 patients with PDD) were calculated for the age at CI, course of disease, C-MMSE, MOCA, ADL, and CDR. P-values mean the comparations between men and women by Mann-Whitney *U* test or chi-squared test in each group. P1 shows the difference of comparation between men and women in MCI-LB group, P2 shows the difference of comparation between men and women in DLB group, P3 shows the difference of comparation between men and women in PD-MCI group, and P4 shows the difference of comparation between men and women in PDD group.

Abbreviation: SD, standard deviation; CI, cognitive impairment; PARK, parkinsonism; T2DM, type 2 diabetes mellitus; APOE, Apolipoprotein E; MTA, medial temporal lobe atrophy; C-MMSE, the Mini-Mental State Examination (Chinese version); MoCA, the Montreal Cognitive Assessment; ADL, the Activity of Daily Living Scale; CDR, the clinical dementia rating; NPI, the Neuropsychiatric Inventory.
